# A Video Based Fire Smoke Detection Using Robust AdaBoost

**DOI:** 10.3390/s18113780

**Published:** 2018-11-05

**Authors:** Xuehui Wu, Xiaobo Lu, Henry Leung

**Affiliations:** 1 School of Automation, Southeast University, Nanjing 210096, China; xhwu@seu.edu.cn; 2 Key Laboratory of Measurement and Control of Complex Systems of Engineering, Ministry of Education, Southeast University, Nanjing 210096, China; 3 Department of Electrical and Computer Engineering, University of Calgary, 2500 University Dr N.W., Calgary, AB T2N 1N4, Canada; leungh@ucalgary.ca

**Keywords:** fire smoke detection, video based, feature extraction, robust AdaBoost, classifier

## Abstract

This work considers using camera sensors to detect fire smoke. Static features including texture, wavelet, color, edge orientation histogram, irregularity, and dynamic features including motion direction, change of motion direction and motion speed, are extracted from fire smoke to train and test with different combinations. A robust AdaBoost (RAB) classifier is proposed to improve training and classification accuracy. Extensive experiments on well known challenging datasets and application for fire smoke detection demonstrate that the proposed fire smoke detector leads to a satisfactory performance.

## 1. Introduction

Detection fire smoke at the early stage has drawn a lot of attentions recently due to its importance to social security and economic development. Conventional point fire smoke detector sensors are effective for indoor applications, but they have difficulties to detect smoke in large outdoor areas, it is because point fire smoke detector typically detect the presence of certain particles generated by smoke and fire by ionization [1], photometry [2], or smoke temperature [3,4]. They require a close proximity to fire and smoke, which are not effective for open spaces. For particles to reach these sensors to activate alarms, many sensors are needed to cover a large area which is not cost effective.

Video based fire smoke detection using cameras is of great interest in large and open spaces [5]. Closed circuit television (CCTV) surveillance systems are widely installed in many public areas to date. These systems can be used to provide early fire smoke detection if a reliable fire detection software is installed in the system. Gottuk et al. [6] test three commercially available video based fire detection systems against conventional spot systems in a shipboard scenario. Video based systems are found to be more effective in flame detection. These systems are economically viable as CCTV cameras are already available for traffic monitoring [7] and surveillance [8] applications. Braovic, M et al. [9] propose an expert system for fast segmentation and classification of regions on natural landscape images that is suitable for real-time automatic wildfire monitoring and surveillance systems. It is noted that smoke is always visible before fire in most outdoor scenarios. This motivates us to research on detecting smoke in the absence or presence of flame from a single frame of video.

There are some technical challenges in video based fire smoke detection. First, it is observed to be inferior to particle-sampling based detectors in terms of false alarm rate. It is mainly due to the variability in smoke density, scene illumination, interfering objects. Second smoke and fire are difficult to be modeled, most of the existing image processing methods do not characterize smoke well [10]. Current fire detection algorithms are based on the use of static and motion information in video to detect flames [11,12,13,14,15]. Many efforts have been made to reduce the false alarm rate and missing detection rate. Table 1 shows some static and dynamic features used in these approaches. As shown, the most used features are color, texture, energy and dynamic features.

On the other side, for classification, some researchers [15,16,18,19,20,21,22] use thresholds or parameters from analysis of extracted features, which is less time-consuming but not adaptive in some complex environments. Some other researchers [30,31] use well known AdaBoost to training models and classify. AdaBoost based component classifiers can solve the overfitting problem relatively with high precision, one weak classifier of component classifiers is a learner which can return a hypothesis that roughly outperform random guessing. For the component classifiers, the weights updates of them in every step mainly depend on the last errors,

(1) $$\alpha_{m}=\frac{1}{2}ln\frac{1-e_{m}}{e_{m}},$$

$\alpha_{m}$ gives the weights of component classifiers, and $e_{m}$ is the errors. Definitely, the weights $\alpha_{m}$ should be positive, so the error $e_{m}$ is required to be less than 0.5, and AdaBoost also requires the error not much less than 0.5, so that the boosting function can make sense of these cascade classifiers. Therefore, we must select a series of proper base classifiers and set a best parameter for every base classifier prudently and it is also time-consuming.

Deep learning is also considered for smoke fire detection. In [32], a binary classifier is trained using annotated patches from scratch, second, learning and classification using cascade convolutional neural network (CNN) fire detector. Muhammad et al. [33] propose an adaptive prioritization mechanism for fire smoke detection. CNN and the internet of multimedia things (IoMT) for disaster management are used for early fire detection framework. Through deep learning, features can be learned automatically. However, these deep learning methods for fire smoke detection are still limited by learning static features. Wu et al. [34] combine deep learning method and conventional feature extraction method to recognize the fire smoke areas. CNN is used in Caffe framework to achieve a Caffemodel with static features. This deep learning approach can work well to certain extent, but the dynamic feature is not trained directly for most situations.

As for fire smoke classification, thresholds or parameters from extracted features are usually used in classification which is less time-consuming. However, it is not adaptive to deal with complex environments [15,16,18,19,20,21,22]. SVM and AdaBoost have been considered for classifier in [23,24,25,26,27,28,29]. SVM can achieve good accuracy with a sufficiently large training set. Some kernel functions can make it possible to solve the nonlinear problem in high dimensional space [35,36,37,38]. One popular kernel used in SVM is RBF (RBFSVM). RBFSVM determines the number and location of the centers and the weight values automatically [39]. It uses a regularization parameter, C, to balance the model complexity and accuracy. Another parameter known as Gaussian width, σ, can be used to avoid the over-fitting problem. However, it is difficult to select proper values of C and σ. AdaBoost based component classifiers can solve the overfitting problem with relatively high precision. One weak classifier of component classifiers is a learner which can return a hypothesis that roughly outperform random guessing.

In this paper, static features, include texture, wavelet, color, hog, irregularity, and dynamic features, include motion direction, change of motion direction and motion speed, are extracted and trained with different combinations. A robust AdaBoost (RAB) classifier is designed for detection. It can overcome the weights update problem and solve the dilemma between high accuracy and over-fitting. RBFSVM (Radial Basic Function—Support Vector Machine) [35] is chosen as the base classifier with some improvements made to guarantee the validity and accuracy of the boosting function. An effective algorithm for locating the original fire position is introduced in this paper for practical fire smoke detection.

The remainder of this article is organized as follows: Video based fire smoke detection using camera are described in Section 2, including static feature in Section 2.1 and dynamic feature extraction in Section 2.2. The proposed Robust AdaBoost classifier for fire smoke detection is presented in Section 3. Performance of the proposed method are evaluated by extensive experiments in Section 4. The paper is concluded in Section 5.

## 2. Features Extraction

**Figure 1:**
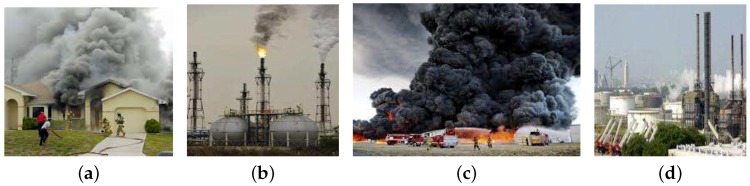
(**a**–**d**) show the fire and smoke image data sets with none-smoke objects.

Fire and smoke training data usually come from pre-collected image datasets [40]. Due to the difficulty in collecting this type of data, these sets are limited and most of them include other random information besides fire smoke as shown in Figure 1. In [29,34], image data sets are used for static feature extraction and set threshold values for classifying dynamic features in real-time video detection. Although this approach can work well to some extent, it is difficult to set a single threshold value that works for situation.

In this paper, different smoke and non-smoke videos taken from camera are used as training samples. Motion regions of all video sequences are used for static feature extraction, and dynamic features are extracted depending on the correlations among successive frames. These training videos are collected and preprocessed so that the videos contain only fire smoke moving objects.

**Figure 2:**
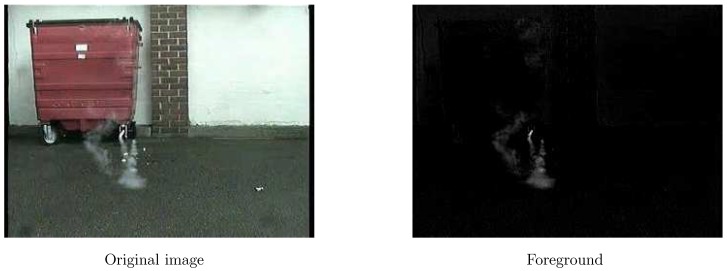
Foreground segmentation.

Robust Orthonormal Subspace Learning (ROSL) [41] method is used to segment foregrounds from images.

(2) $$\min_{D,\alpha ,E}{∥\alpha ∥}_{row-1}+\lambda {∥E∥}_{1},s.t\;D\alpha +E=M,A=D\alpha ,D^{T}D=I_{k},\forall i,$$

where *M* denotes the observed matrix of video, *A* is the low-rank background matrix, and *M* is the foreground matrix. This method represents *A* under the ordinary orthonormal subspace $D\;=\;\left[D_{1},D_{2},\ldots ,D_{k}\right]\in R^{m\times n}$, where coefficients $\alpha =\left[\alpha_{1};\alpha_{2};\ldots ;\alpha_{k}\right]\in R^{k\times n}$. The dimension *k* of the subspace is set as k=βr (β is a constant and β>1). *E* is the sparse matrix which represents foregrounds. Foreground segmentation results are shown in Figure 2. Motion regions are selected frame by frame. In order to choose the exact sample data sets that we need, tiny motion regions which may be created by minute jitter or too big ones should be eliminated.

### 2.1. Static Features Extraction

Color, texture and energy in wavelet domain are three commonly used static features as shown in Table 1. Here we consider three other static features including Edge Orientation Histogram (EOH) [42], irregularity and sparsity, and three dynamic features including motion direction, change of motion direction and motion speed.

#### 2.1.1. Color, Texture and Energy

Color moments are measures that can be used to differentiate images. Stricker and Orengo [43] propose three central moments of an image color distribution such as mean, standard deviation and skewness. Here we use HSV (Hue, Saturation, and Value) to define three moments for each three color channels: mean, standard deviation and skewness of the *i*th color channel at the *j*th image pixel $p_{ij}$:

(3) $$E_{i}=\frac{1}{N}\sum_{j=1}^{N}p_{ij}$$

(4) $$\sigma_{i}=\sqrt{\left(\frac{1}{N}\sum_{j=1}^{N}{\left(p_{ij}-E_{i}\right)}^{2}\right)}$$

(5) $$s_{i}=\sqrt[{3}]{\left(\frac{1}{N}\sum_{j=1}^{N}{\left(p_{ij}-E_{i}\right)}^{3}\right)}$$

LBP feature has been used to capture spatial characteristics of an image as reported in [22,44]. The LBP features are extracted as texture descriptor of smoke in this study. When smoke is found in a region, the edge of background will be blurred so that the high frequency energy of this region will decrease. Gabor wavelet is used to get three high frequency components in horizontal (HL), vertical (LH) and diagonal (HH) directions. The energy of image in region $R_{i}$ is defined as

(6) $$EN_{i}=\frac{\sum_{\left(x,y\right)\in R_{i}}w\left(x,y\right)}{\sum_{\left(x,y\right)\in R_{i}}W\left(x,y\right)},$$

where $EN_{i}$ is the energy of image in region $R_{i}$ and $w\left(x,y\right)$ is high frequency wavelet coefficients of pixel at (x,y) in region $R_{i}$ and $w\left(x,y\right)$ is the entire wavelet coefficients of the same pixel. We have

(7) $$w\left(x,y\right)={\left|HL\left(x,y\right)\right|}^{2}+{\left|LH\left(x,y\right)\right|}^{2}+{\left|HH\left(x,y\right)\right|}^{2},$$

(8) $$W\left(x,y\right)={\left|HL\left(x,y\right)\right|}^{2}+{\left|LH\left(x,y\right)\right|}^{2}+{\left|HH\left(x,y\right)\right|}^{2}+{\left|LL\left(x,y\right)\right|}^{2}.$$

#### 2.1.2. Edge Orientation Histogram

Gradients of an image plays an important role in object detection. The Scale Invariant Feature Transform (SIFT) [45] achieves an impressive performance on image registration and object detection. As a variant of SIFT, Histograms of Oriented Gradients (HOG) [46] are used for human detection. HOG can capture local gradient-orientation structures within an image but it is computed on a dense grid of uniform space over an image. Therefore, a high dimensional feature vector is produced to describe each detection window, which is not suitable for real-time application like fire smoke detection. Levi and Weiss [42] propose the Edge Orientation Histogram (EOH) method which measures the response of linear edge detectors at different subareas of the input image.

In this paper, we use Canny operator to generate image edge and the gradient which contains horizontal component $G_{x}$ and vertical component $G_{y}$. The edge gradient $\left[G_{x},G_{y}\right]$ is then converted into polar coordinate. That is

(9) $$m\left(x,y\right)=\sqrt{G_{x}^{2}+G_{y}^{2}},$$

(10) $$\theta \left(x,y\right)=\tan^{-1}\left(\frac{G_{y}}{G_{x}}\right),$$

where *m* is the magnitude and θ denotes the orientation.

The edge orientations are mapped to the range $\left[-180^{{}^{\circ}},180^{{}^{\circ}}\right]$ with an interval of 10°. The numbers of edge orientation in these 36 angle ranges are counted using two angle threshold matrices as $T_{1}=\left[-170^{{}^{\circ}},-160^{{}^{\circ}},\;\ldots ,160^{{}^{\circ}},170^{{}^{\circ}},180^{{}^{\circ}}\right]$ and $T_{2}=\left[-180^{{}^{\circ}},-170^{{}^{\circ}},\ldots ,150^{{}^{\circ}},160^{{}^{\circ}},170^{{}^{\circ}}\right]$,

(11) $$h\left(k\right)\left\{\begin{array}{c} +1,\theta \left(x,y\right)\in \left[T_{2}\left(k\right)T_{1}\left(k\right)\right)\\ +0,\mathrm{otherwise} \end{array}\right.,k=1,2,\ldots ,36.$$

For each angle range $\left[T_{2}\left(k\right),T_{1}\left(k\right)\right)$, the EOH is obtained by summing all the gradient magnitudes whose orientations belongs to this range,

(12) $$E\left(k\right)=\sum_{\theta \left(x,y\right)\in \left[T_{2}\left(k\right),T_{1}\left(k\right)\right]}m\left(x,y\right)$$

The EOH features can be extracted with two different methods- Dominant Orientation Features (DOF) and Symmetry Features (SF) [42]. When we try to find the dominant edge orientation in a specific area rather than the ratio between two different orientations, we define a slightly different set of features which measures the ratio between a single orientation and the others. That is

(13) $$E_{k,dof}=\frac{E\left(k\right)+\varepsilon }{\sum_{k=1}^{36}E\left(k\right)+\varepsilon },k=1,2,\ldots ,36,$$

where $E_{k,dof}$ records the ratio of every single orientation from $E_{k,dof}$ to $T_{1}\left(k\right)$, ε is a tiny positive value to avoid division by zero. According to Equation (7), the domain orientation is located in $\left[T_{2}\left(k_{d}\right),T_{1}\left(k_{d}\right)\right)$.

According to [42], we define the symmetry features in $\left[T_{2}\left(k\right),T_{1}\left(k\right)\right)$ as

(14) $$E_{k,sf}=\left\{\begin{array}{c} \frac{\left|R_{1}\left(k\right)-R_{2}\left(k\right)\right|}{h\left(k\right)},h\left(k\right)\neq 0\\ \max\left(E_{k,sf}\right),h\left(k\right)=0 \end{array}\right.,$$

To normalize this feature, we reset it as

(15) $$E_{k,sf}=\frac{E_{k,sf}}{\max\left(E_{k,sf}\right)},$$

where $\left(R_{1}\left(k\right),R_{2}\left(k\right)\right)=\left\{\begin{array}{c} \left(E\left(k\right),E\left(18+k\right)\right),k\in \left[1,18\right]\\ \left(E\left(k\right),E\left(k-18\right)\right),k\in \left[19,36\right] \end{array}\right.$, $R_{1}$ and $R_{2}$ are rectangles of the same size and are positioned at opposite sides of the symmetry axes. Not only the symmetry features can be used to find symmetry, but it can also find places where symmetry is absent. From Equation (9), a small $E_{k,sf}$ means that the image orients more in $\left[T_{2}\left(k\right),T_{1}\left(k\right)\right]$ compared to its opposite side range.

The symmetry value of the entire detected region is computed as

(16) $$E_{SF}=\frac{\left|2\sum_{k=k_{s}}^{17+k_{s}}E_{k,sf}-\sum_{k=1}^{36}E_{k,sf}\right|+\varepsilon }{\sum_{k=1}^{36}E_{k,sf}+\varepsilon },$$

(17) $$\begin{array}{c} k_{s}=\underset{k^{*}}{\arg\min}\left|\sum_{k=k^{*}}^{17+k^{*}}E_{k,sf}-\left(\sum_{k=1}^{36}E_{k,sf}-\sum_{k=k^{*}}^{17+k^{*}}E_{k,sf}\right)\right|\\ =\underset{k^{*}}{\arg\min}\left|2\sum_{k=k^{*}}^{17+k^{*}}E_{k,sf}-\sum_{k=1}^{36}E_{k,sf}\right|,k^{*}\in \left[1,18\right] \end{array}$$

$E_{sf}$ is small when the detected motion region is symmetric.

**Figure 3:**
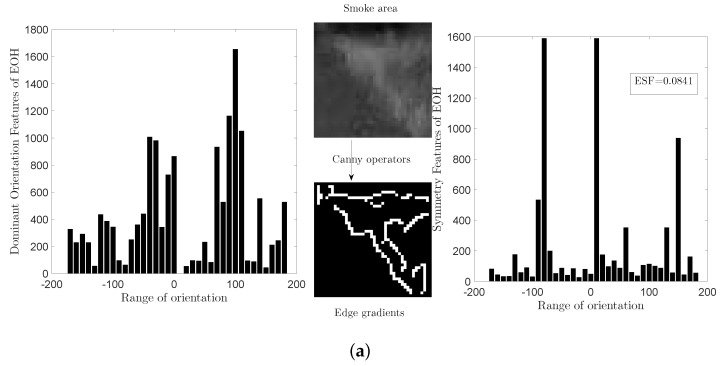

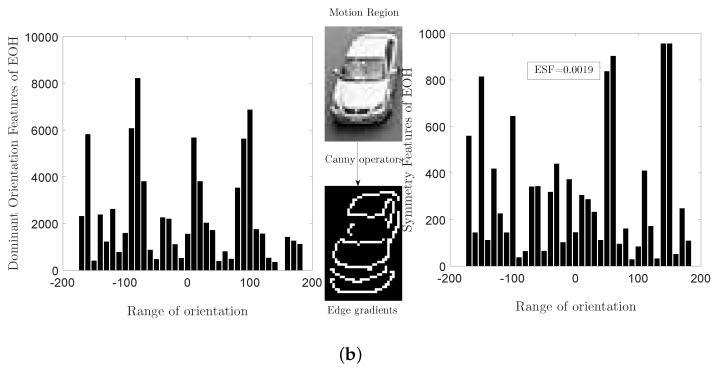
Histogram of Edge Orientation: Left figure of (**a**,**b**): Dominant Orientation Features. Right figure of (**a**,**b**): Symmetry Features.

Figure 3 shows the two different EOH features ($E_{dof}$ and $E_{sf}$) of smoke and car images. In this paper, we concatenate these two EOH features to characterize the edge features.

#### 2.1.3. Irregularity and Sparsity

Fire smoke have no regular shapes in diffusion, this irregularity feature is defined as

(18) $$IR=\frac{c^{2}}{4\pi s}$$

where IR is the irregularity, *c* is the edge perimeter of detected region and *s* is the area.

**Figure 4:**
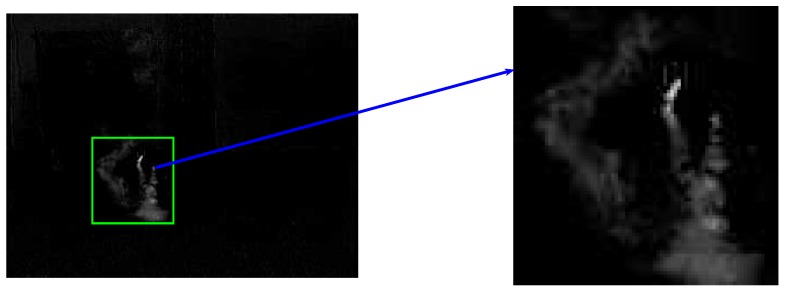
Sparsity of foreground.

For the sparsity feature, it is mainly for light smoke as described in [22]. We extract the sparse foreground with ROSL [41], and *E* in Equation (2) is the foreground matrix. Figure 4 shows the extracted sparse smoke without backgrounds.

The sparsity value is calculated as

(19) $$SP=\frac{{∥F_{M}∥}_{0}}{S_{F_{M}}}$$

where $F_{M}$ is the matrix of region *M*, $S_{F_{M}}$ is the size of $F_{M}$. Figure 4 shows the foreground motion region. The ℓ1 norm counts the numbers of non-zero pixels in foreground.

### 2.2. Dynamic Features Extraction

**Figure 5:**
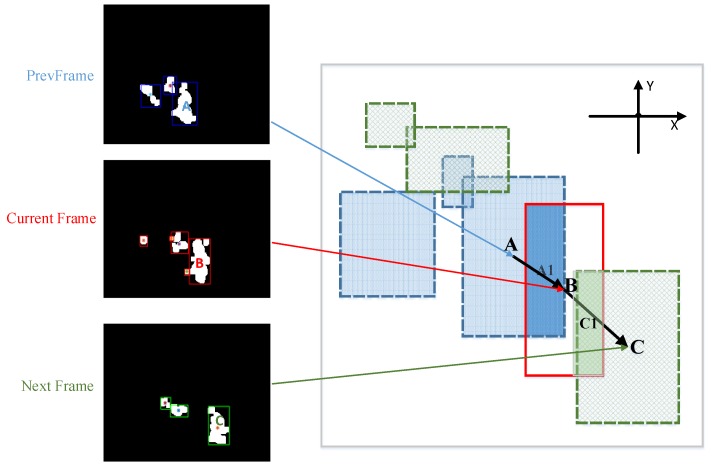
Overlapping ratio computing.

Fire smoke has special dynamic features that provides information for recognition. Motion direction, change of direction and motion speed are extracted as three dynamic features. Before extracting these features, we need to find the locations of the detected motion region in consecutive frames. The centroid of current motion region is first calculated. Because a moving object in two adjacent frames has similar location and shape, these two motion regions have maximum overlap ratio and the shortest distance. Figure 5 shows the mechanism of finding the locations of these two corresponding motion regions. In Figure 5, A, B and C are the centroid of three successive frames, A1 and C1 are the two overlapping regions, region A has the maximum overlap ratio computed with current detected region B for the last frame while region C has the maximum overlapping ratio computed with current detected region B for the next frame, the overlapping ratio is calculated as

(20) $$OVR_{Last}=\frac{S_{A_{1}}}{S_{A}+S_{B}-S_{A_{1}}},OVR_{next}=\frac{S_{C_{1}}}{S_{A}+S_{C}-S_{C_{1}}}$$

where S. represents the area of each region shown in the figure.

From Figure 5, motion region B is detected moving from A to C in three consecutive frames. Hence, its motion direction can be calculated with the orientation of $\vec{AB}$ and $\vec{BC}$. Define the horizontal component of $\vec{AB}$ as $f_{X-AB}$ and the vertical component of $\vec{AB}$ as $f_{X-AB}$, the motion direction of region B is calculated as

(21) mdA=tan−1fX−ABfX−ABfY−ABfY−AB,

As smoke moves slowly and its motion is not obvious in adjacent two frames, we use the statistic measurement to extract the motion features by calculating the mean values of corresponding motion region directions from $t_{1}$ and $t_{2}$ instead of that in adjacent two frames. The final motion direction feature of one motion region in frame $t_{1}$ is defined by

(22) $$MD_{t1}=\frac{\sum_{i=t_{1}}^{t_{2}}md_{i}}{t_{2}-t_{1}}.$$

We set the frame interval as $t_{2}-t_{1}=5$. Each motion region can be calculated by following the scheme above (MD in the first four frames and last four frames cannot be calculated), such that we can also obtain one motion direction change in frame $t_{1}$

(23) $$mdc_{t1}=md_{t1}-md_{t2},$$

Another dynamic feature is called motion speed, while we can easily achieve the motion speed with the distance of two corresponding motion regions in consecutive two frames as

(24) $$ms_{A}=\left|\vec{AB}\right|\times R_{f}.$$

where $E_{f}$ is the frame rate, the motion speed from $t_{1}$ to $t_{2}$ is calculated as

(25) $$MS_{t1}=\frac{\sum_{i=t_{1}}^{t_{2}}ms_{i}}{t_{2}-t_{1}}.$$

Combining different features can enhance the robustness of features. According to [24], features can be concatenated together to form a feature vector in cascade fusion as follows:

(26) $$FV_{i}=\left\{F_{i}^{1},F_{i}^{2},\ldots ,F_{i}^{n},\right\}$$

where $F_{i}^{n}$ represents every single features of one image.

**Figure 6:**
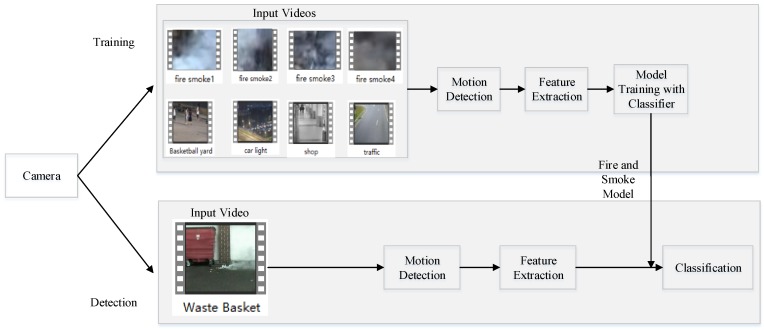
Framework of fire smoke detector.

Figure 6 shows the framework of fire smoke detection in this paper including motion region detection, feature extraction and samples training and fire smoke classification. As for the classifier, we propose a robust AdaBoost method in the following section.

## 3. The Proposed Robust AdaBoost Classifier for Fire Smoke Detection

### 3.1. AdaBoost

Given a set of samples $s={\left\{\left(x_{i},y_{i}\right)\right\}}_{i=1}^{N}$, x∈χ, χ represents the input space, and $\chi \subset R^{N}$, $y\in \left\{-1,+1\right\}$ is the label. The goal of AdaBoost is to train a series of weak classifiers to be a stronger one. Boosting algorithms improve the performance of a learning algorithm by calling a weak learner in a succession of cycles, in each cycle, it maintains a weight distribution $D_{m}$ over the weak learner with input set *S* and returns a hypothesis $h_{m}$. The weight distribution is initially set uniform as $D_{1}$,

(27) $$D_{1}=\left(w_{11},w_{12},\ldots ,w_{1i},\ldots ,w_{1N}\right),w_{1i}=\frac{1}{N},i=1,2,\ldots ,N,$$

We use the distribution $D_{m}$ to train the base classifier $G_{m}\left(x\right)$ with training dataset and get errors $e_{m}$,

(28) $$e_{m}=P\left(G_{m}\left(x_{i}\right)\neq y_{i}\right)=\sum_{i=1}^{N}w_{mi}I\left(h_{mi}\neq y_{i}\right)\in \left[0,1\right].$$

The weight of weak classifier $\alpha_{m}$ can be achieved with error $e_{m}$ by $\alpha_{m}=\frac{1}{2}ln\frac{1-e_{m}}{e_{m}}$, when $e_{m}\leq \frac{1}{2}$, $\alpha_{m}\geq 0$, and $\alpha_{m}$ increases with the decrease of $e_{m}$. That is, the smaller the classification error is, the more important the role of this weak classifier is of the component classifiers. For the next cycle, the weights of samples are updated as

(29) $$D_{m+1}=\left(w_{m+1,1},w_{m+1,2},\ldots ,w_{m+1,i},\ldots ,w_{m+1,N}\right),$$

(30) $$w_{m+1,i}=\frac{w_{mi}}{Z_{m}}e^{\left(-\alpha_{m}y_{i}G_{m}\left(x_{i}\right)\right)},\left(w_{0,i}=\frac{1}{N}\right)$$

(31) $$Z_{m}=\sum_{i=1}^{N}w_{mi}e^{\left(-\alpha_{m}y_{i}G_{m}\left(x_{i}\right)\right)},$$

where $Z_{m}$ is the normalization factor. This distribution $D_{m+1}$ shows that the weights of the misclassified samples will be increased and the weight of the correctly classified samples will be reduced. In this way, AdaBoost will be forced to pay its attention to the samples that have been misclassified in the next cycle.

Combine all component weak classifiers,

(32) $$f\left(x\right)=\sum_{m=1}^{M}\alpha_{m}G_{m}\left(x\right),$$

where *M* is the total number of cycles. The final classifier is defined as

(33) $$G\left(x\right)=sign\left(f\left(x\right)\right),$$

AdaBoost calls this algorithm repeatedly in a series cycles and can get a lower training error with a higher accuracy.

### 3.2. Proposed Robust AdaBoost

One important theoretical property of AdaBoost is that component classifiers need to be only slightly better than random guessing, the weight $\alpha_{m}$ will be negative if $e_{m}>0.5$ and be larger than 1 when $e_{m}<\frac{1}{e^{2}+1}$, that means we should spend time on selecting moderate parameter so that the error created by this weak classifier is slightly less than 0.5, otherwise, the classifier with classification error slightly more than 0.5 will be discarded. When the weak classifier error is less than 0.1, its weight will be relatively high, it can just take control of errors in a tiny limited range. However, AdaBoost treat this limited controlled errors as the only key for updating weights. Either too strong or too weak a classifier will not satisfy the boosting requirements. The weight calculation of base classifier is modified as

(34) $${\alpha_{m}}^{*}={\left(\ln\frac{2e}{1+e^{e_{m}}}\right)}^{n},n\geq 1.$$

**Figure 7:**
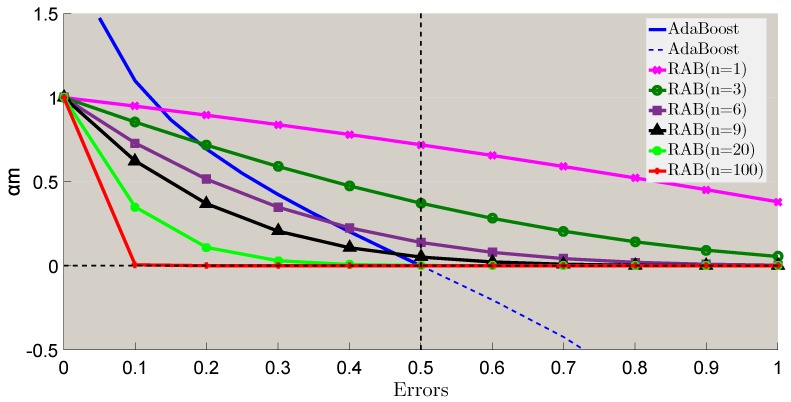
Base classifier weights update.

As $e_{m}\in \left[0,1\right]$,${\alpha_{m}}^{*}\in \left[{\left(\ln\frac{2e}{1+e}\right)}^{n},1\right]$, $\ln\frac{2e}{1+e}\approx 0.38$. Figure 7 shows these two weight computing methods of conventional AdaBoost and robust AdaBoost in this paper. ${\alpha_{m}}^{*}$ also decrease with an increasing $e_{m}$. No matter how weak or strong the base classifier is, it can achieve its corresponding weight. The stronger the base classifier is, the more important it will be. While *n* is large enough, the weight of the base classifier that slightly outperforms random guessing ($e_{m}\approx 0.5$) will almost equal to zero. When $e_{m}$ approaches to zero, the weight ${\alpha_{m}}^{*}$ approaches to zero. Therefore, our proposed method is consistent with boosting theory.

AdaBoost can decrease its training errors through its learning. Its error upper bound is given by

(35) $$E=\frac{1}{N}\sum_{i=1}^{N}I\left(G\left(x_{i}\right)\neq y_{i}\right)\leq \frac{1}{N}\sum_{i=1}^{N}\exp\left(-y_{i}f\left(x_{i}\right)\right),$$

when $G\left(x_{i}\right)\neq y_{i}$, $y_{i}f\left(x_{i}\right)\leq 0$, $\exp\left(-y_{i}f\left(x_{i}\right)\right)\geq 1$. According to Equations (30), (31) and (35), the error upper bound can be calculated as

(36) $$\begin{array}{c} E=\frac{1}{N}\sum_{i=1}^{N}\exp\left(-y_{i}f\left(x_{i}\right)\right)=\frac{1}{N}\sum_{i=1}^{N}\exp\left(-\sum_{m=1}^{M}\alpha_{m}y_{i}G_{m}\left(x_{i}\right)\right)\\ =w_{1i}\sum_{i=1}^{N}\exp\left(-\sum_{m=1}^{M}\alpha_{m}y_{i}G_{m}\left(x_{i}\right)\right)\\ =w_{1i}\prod_{m=1}^{M}\exp\left(-\alpha_{m}y_{i}G_{m}\left(x_{i}\right)\right)\\ =Z_{1}\sum_{i=1}^{N}w_{2i}\prod_{m=2}^{M}\exp\left(-\alpha_{m}y_{i}G_{m}\left(x_{i}\right)\right)\\ =Z_{1}Z_{2}\sum_{i=1}^{N}w_{3i}\prod_{m=3}^{M}\exp\left(-\alpha_{m}y_{i}G_{m}\left(x_{i}\right)\right)\\ =Z_{1}Z_{2}\cdots Z_{M-1}\sum_{i=1}^{N}w_{Mi}\left(-\alpha_{M}y_{i}G_{M}\left(x_{i}\right)\right)\\ =\prod_{m=1}^{M}Z_{m} \end{array},$$

The error upper bound of the component classifiers is $\prod_{m=1}^{M}Z_{m}$. It means that we can minimize $Z_{m}$ by selecting moderate base classifier in every cycle, and make the training errors decrease moderately.

When it comes to binary classification, Equation (32) can be written as

(37) $$\begin{array}{cc} Z_{m} & =\sum_{y_{i}=G_{m}\left(x\right)}w_{mi}e^{-\alpha_{m}}+\sum_{y_{i}\neq G_{m}\left(x\right)}w_{mi}e^{\alpha_{m}}\\ & =\left(1-e_{m}\right)e^{-\alpha_{m}}+e_{m}e^{\alpha_{m}}\\ & =2\sqrt{e_{m}\left(1-e_{m}\right)} \end{array}$$

For the proposed Robust AdaBoost,

(38) $$\begin{array}{c} {Z_{m}}^{*}=\sum_{y_{i}=G_{m}\left(x\right)}w_{mi}e^{-{\alpha_{m}}^{*}}+\sum_{y_{i}\neq G_{m}\left(x\right)}w_{mi}e^{{\alpha_{m}}^{*}}\\ =\left(1-e_{m}\right)e^{-{\alpha_{m}}^{*}}+e_{m}e^{{\alpha_{m}}^{*}}\\ =\left(1-e_{m}\right)e^{-{\left(\ln\frac{2e}{1+e^{e_{m}}}\right)}^{n}}+e_{m}e^{{\left(\ln\frac{2e}{1+e^{e_{m}}}\right)}^{n}}\\ =\left(1-e_{m}\right)e^{-{\left(\ln\frac{1+e^{e_{m}}}{2e}\right)}^{n}}+e_{m}e^{{\left(\ln\frac{2e}{1+e^{e_{m}}}\right)}^{n}} \end{array}.$$

**Figure 8:**
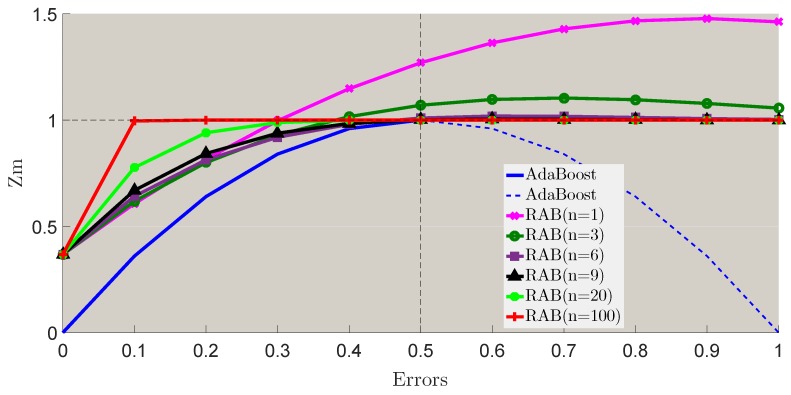
Weights normalization values of AdaBoost.

Figure 8 shows the different $Z_{m}$ changes with different errors of conventional AdaBoost and the proposed robust AdaBoost (*n* = 1, 3, 6, 9, 20, 100). Definitely, the goal of boosting is to achieve a series base classifiers with lower errors. When we focus on the $Z_{m}$ with error less than 0.5, it is clear that the proposed robust AdaBoost is consistent with the boosting function, Furthermore, it is also appropriate for the entire domain of definition [0,1], which is more robust than conventional AdaBoost.

### 3.3. Feasibility Analysis for Choosing RBFSVM as Base Classifier

This paper aims at applying RBFSVM as component classifier in boosting. There are two vital parameters, Gaussian width σ and regularization parameter *C*, that are relevant to classification performance. The classification performance of RBFSVM can mainly depend on σ value with a roughly suitable *C* [47]. We can adjust RBFSVM to be an appropriate base classifier of boosting component classifiers by simply changing σ over a large range of *C*.

In [48], σ uses a large initial value $\sigma_{ini}=12$ and decreases it slightly to increase the learning capacity of RBFSVM so that its error is less than 50%. In this paper, instead of adjusting errors to be less than 0.5, we focus on how to make these classifiers uncorrelated and improve the performance of every base classifier. Because of the robustness described in Section 3, our weight computing method can keep balance with errors in [0,1].

#### 3.3.1. Diversity

In order to make the boosting function more efficient for the component classifiers, a roughly large σ is set initially. There is another important factor affecting the generation performance of ensemble methods called Diversity [49,50]. It is also appropriate in AdaBoost, so that these component classifiers have irrelevance and the boost function can make sense of boosting these same classifiers with different properties.

Similar to [48], the diversity is calculated as follows: If $h_{m,i}$ is the hypothesis of the *m*th SVM classifier on the sample $x_{i}$, and $f\left(x_{i}\right)$ is the combined prediction label of all the existing component classifiers, the diversity of the *m*th component classifier on the sample $x_{i}$ is calculated as

(39) $$d_{mi}=\left\{\begin{array}{c} 0,ifI\left(h_{mi}=f\left(x_{i}\right)\right)=1\\ 1,ifI\left(h_{mi}=f\left(x_{i}\right)\right)=0 \end{array}\right.,$$

and the diversity value of RAB-RBFSVM in *m* th cycle for *N* samples is

(40) $$DIV_{m}=\frac{1}{N}\sum_{i=1}^{N}d_{mi}.$$

Here we do not compare $DIV_{m}$ with a preset threshold, as it is hard to find a single threshold for different training data. σ is decreased step by step to find a proper new $\sigma^{*}$ that can minimize the error and maximize the diversity simultaneously. The step length of the decreasing σ is

(41) $$\sigma_{step}=\frac{\sigma_{ini}}{50}.$$

#### 3.3.2. Samples Processing: Denoising Initial Weights

With the update of σ, several RBFSVM classifiers using different σs are tested on the same samples in one cycle. From Equation (30), we know that the weights of misclassified samples will be increased. However, if a sample is misclassified too many times in one cycle, there is a large possibility that this sample is a noise of this training data, and its weight should be decreased relatively. The misclassified times of the sample should be inversely proportional to its weight, and the weights of misclassified samples as follows:

(42) $$I_{m}=\left|I_{tr}-I_{tr,\max}\right|,$$

(43) $$I_{m\_norm}=\frac{I_{m}}{{∥I_{m}∥}_{2}},\left(I_{m\_norm}\in \left(0,1\right]\right)$$

(44) $$w_{m,I_{m}}=\ln\frac{2e}{1+e^{I_{m\_norm}}},\left(w_{m,I_{m}}\in \left[\ln\frac{2e}{1+e},1\right)\right)$$

(45) $$w_{m+1,i}=w_{m,I_{m}}\frac{w_{mi}}{Z_{m}}e^{\left(-\alpha_{m}^{*}y_{i}G_{m}\left(x_{i}\right)\right)}.$$

where $I_{tr}$ is the total times of misclassification in one cycle, and $I_{tr,\max}$ is the max value, Equations (42)–(44) calculate the possibility of classification of each sample $w_{m,I_{m}}$. $w_{m+1,i}$ is the final weight of samples in each cycle.

The conventional AdaBoost starts its boost with the same weights of samples, which means it does not take the balance of negative and positive samples into consideration in the first step. In RAB-RBFSVM+, the initial weights are calculated with different scales of negative and positive samples. For binary classification,

(46) $$w_{1i}^{n}=\frac{1}{2N_{n}},w_{1i}^{p}=\frac{1}{2N_{p}},i=1,2,\cdots ,N$$

$N_{n}$ is the number of negative samples and $N_{p}$ positive, $w_{1i}^{n}$ and $w_{1i}^{p}$ are the initial weights of negative and positive samples. Algorithm 1 shows the entire process of our proposed RAB-RBFSVM+.
**Algorithm 1** Improved Robust AdaBoost classifier with RBFSVM: RAB-RBFSVM+.**Input:** Training datasets $s={\left\{\left(x_{i},y_{i}\right)\right\}}_{i=1}^{m}$ with labels $y\in \left\{-1,+1\right\}$.
1:**initialize:** Normalize training datasets; Initialize weights of datasets via Equation (46), $\sigma_{step}$ via Equation (41); $\sigma_{ini}\leftarrow 12$;$m\leftarrow 1;err\leftarrow -1;N\leftarrow 100;I_{0\_tr}\leftarrow I$;2:**while**m<N&err≠0**do**3: Train a RBFSVM base classifier ht on the weighted training datasets and get the misclassification flag of samples $I_{m}$.4: Compute training errors using Equation (28)5: Compute diversity value using Equations (39) and (40)6: Compute misclassification times of samples: $I_{m\_tr}=I_{m-1\_tr}+I_{m}$7: Compute possibility of noise samples via Equations (42)–(44)8: Decrease σ by $\sigma_{m}$9: **if** $\sigma >\sigma_{step}\;\&\;err\neq 0$ **then**10:  Catch the right σ which minimize the error: $\sigma_{m}$, and go to step 311: **end if**12: $\sigma =\sigma_{m}$13: Repeat step 3-714: Set the weight of component classifier via Equation (34)15: Update the weight of samples via Equation (45)16:**end while**
**Output:** Combine classifiers via Equations (32) and (33)

## 4. Experimental Results

In this section, we evaluate the performance of Robust AdaBoost (RAB) and the improved Robust AdaBoost RBFSVM (RAB-RBFSVM+). We compare the proposed algorithm with the conventional AdaBoost based component classifiers using other weak classifiers. The data sets from UCID Repository [51] are utilized to verify the effectiveness of the proposed robust AdaBoost classifier.

Different feature combinations are also compared using different algorithms we test above. The smoke and non-smoke videos are collected from public video clips (The related video clips can be downloaded from http://signal.ee.bilkent.edu.tr/VisiFire/Demo/SampleClips and http://imagelab.ing.unimore.it/visor). The smoke clips in this data sets cover indoor and outdoor with different illumination, short or long distance surveillance scenes. Seven smoke videos and ten non-smoke videos are used in the experiment. In the training datasets of smoke, we select or reprocess the smoke videos so that they only have smoke motion regions. In this paper, we extracted 5699 motion regions (include 2766 positive samples and 2933 negative samples) for training and test. Finally, we test three videos with smoke, non-smoke or mixed sequences.

### 4.1. Advantages of the Proposed Classifier

According Equation (34) and Figure 6, the weight parameter ${\alpha_{m}}^{*}$ has different domain of values with different *n* in $e_{m}\in \left[0,1\right]$, as shown in Table 2.

**Figure 9:**
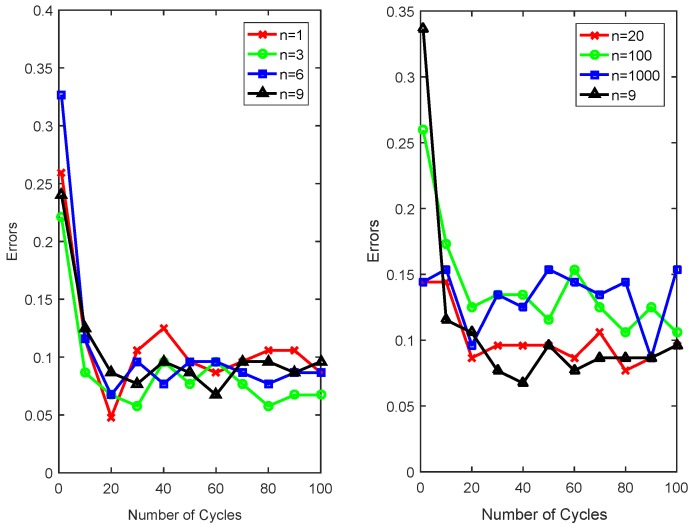
Classification errors using different ${\alpha_{m}}^{*}$ with different *n*s.

Theoretically, we expect that the well-performed classifier with lower errors could have higher weight while the poor one has roughly lower weight, so that the performance of AdaBoost could depend much more on these well performed classifiers, therefore, we appropriate a large *n*. However, we also prefer to have a robust algorithm for classifier with large errors rather than discard it directly, when *n* is too large, ${\alpha_{m}}^{*}$ will close to zero with large error and its computing complexity will increase too, it is unnecessary and not rational to use an extreme large *n*. Table 2 shows the range changes of ${\alpha_{m}}^{*}$ with respect to *n*, when n=1, the ${\alpha_{m}}^{*}$ changes from a relatively large value to 1, and when $e_{m}=0.5$, the value of ${\alpha_{m}}^{*}$ is also large, it is lack of effectiveness for boosting well-performed classifiers with $e_{m}\ll 0.5$. Additionally, when n>6, classifier with large errors will be discarded directly since its weight is too small. These *n*s in Table 2 are tested and the results are shown in Figure 9. When the number of cycles is large, their differences are not very distinct. When *n* is less than 10 (n=1,3,6,9 ) in left subfigure, the right subfigure of Figure 9 demonstrates that the performance degrades when n=100 or n=1000. In this paper, we set n=3.

**Figure 10:**
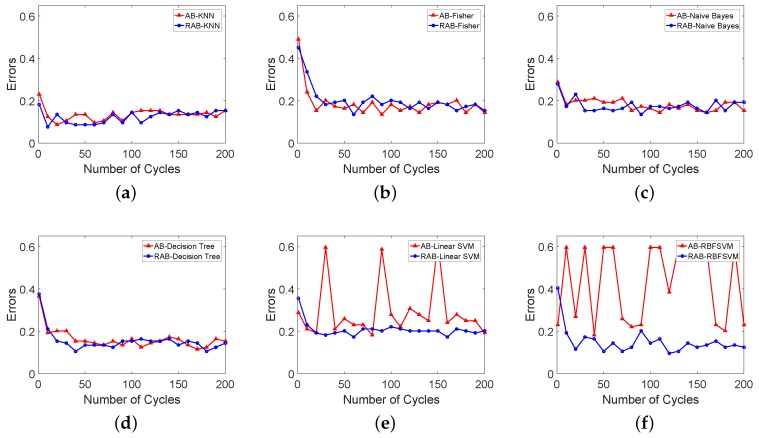
Performances of AdaBoost and Robust AdaBoost with different component classifiers. (**a**) Training errors of AB-KNN and RAB-KNN; (**b**) Training errors of AB-Fisher and RAB-Fisher; (**c**) Training errors of AB-Naive Bayes and RAB Naive Bayes; (**d**) Training errors of AB-Decision Tree and RAB-Decision Tree; (**e**) Training errors of AB-Linear SVM and RAB-Linear SVM; (**f**) Training errors of AB-RBFSVM and RAB-RBFSVM.

The base classifiers are listed as KNN (K-Nearest Neighbor), Fisher Linear Discriminant, Naive Bayes classifier, DTC(decision tree classifier) and SVM (Support Vector Machines), and RBFSVM, there are no further improved methods used except Robust AdaBoost and the initial σ is set as $\sigma_{ini}=12$ for RBFSVM in this section. Figure 10 shows the performances of the two AdaBoost methods. AB represents AdaBoost method, and RAB represents Robust AdaBoost method. The results of the 50th, the 100th, the 150th, and the 200th cycles are listed in Table 3.

For Fisher Linear Discriminant, Robust AdaBoost performs as well as AdaBoost. For Naive Bayes, KNN, and Decision Tree classifiers, the Robust AdaBoost gives a little better boosting performance than AdaBoost, and both of them perform better than a single classifier. For Linear SVM and RBFSVM, the performance of Robust AdaBoost is much better than AdaBoost with lower errors and more stable changes. It is clear that the normal AdaBoost cannot boost SVM classifiers. The proposed RAB gives a better boosting result. Table 3 presents that Robust AdaBoost based RBFSVM component classifiers has the best result and it is also more efficient than the other classifiers without boosting.

**Figure 11:**
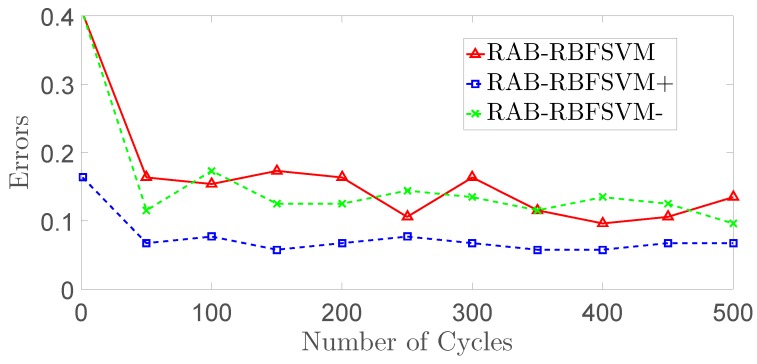
Performances of RAB-RBFSVM and Performances of RAB-RBFSVM, RAB-RBFSVM− and RAB-RBFSVM+

Figure 11 shows the error changes of three different RAB-RBFSVM methods: RAB-RBFSVM without any improvements except the Robust AdaBoost; RAB-RBFSVM with improvements of diversity (RAB-RBFSVM−), and RAB-RBFSVM with both improvements of diversity and sample processing (RAB-RBFSVM+). The results 50th, 100th, 200th, 300th, 400th, 500th cycle are shown in Table 4.

From Figure 10 and Table 4, RAB-RBFSVM− method with diversity performs better than RAB-RBFSVM, and our proposed method, RAB-RBFSVM+ with improvements of diversity and samples processing, achieves the best results with more stable changes and lower errors, which indicates the effectiveness of these improvements for RAB-RBFSVM.

In order to compare the proposed classifier with other different classifiers using common datasets from UCID, we test AdaBoost with KNN (K-Nearest Neighbor)component classifiers, (AB-KNN), AdaBoost with Fisher Linear Discriminant component classifiers, (AB-FLD), AdaBoost with Decision Tree component classifiers, (AB-DT), AdaBoost with Naive Bayes component classifiers, (AB-NB), AdaBoost with Linear SVM component classifiers, (AB-SVM), Diverse AdaBoostSVM, (DAB-SVM), our proposed Robust AdaBoost RBFSVM (RAB-RBFSVM+), in addition to these component classifiers, single RBFSVM classifier and linear SVM classifier are also compared. Table 5 shows the generation errors of different classifiers. Note that the proposed method generates lower errors on the datasets.

### 4.2. Performance Advantages on Fire Smoke Detector

In this section, we will compare our proposed RAB-RBFSVM+ with other methods using our extracted fire smoke features in different combinations. Different static and dynamic features are extracted as described in Section 2. Extensive experiments are carried out to test these features in different combinations, and the effectiveness of our proposed method. For brevity, the static features are simply marked with their initials (Color-‘C’, Texture-‘T’, Wavelet energy-‘W’, Edge orientation histogram-‘E’, irregularity-‘I’ and sparsity-‘S’), dynamic features are marked as ‘MD’ (Motion direction), ‘MDC’ (change of motion direction) and ‘MS’ (motion speed), ‘Static’, ‘Dynamic’ and ‘All’ represent all the static features, dynamic features and all features combinations separately. We divide them into six groups according to the combination rules.

**Figure 12:**
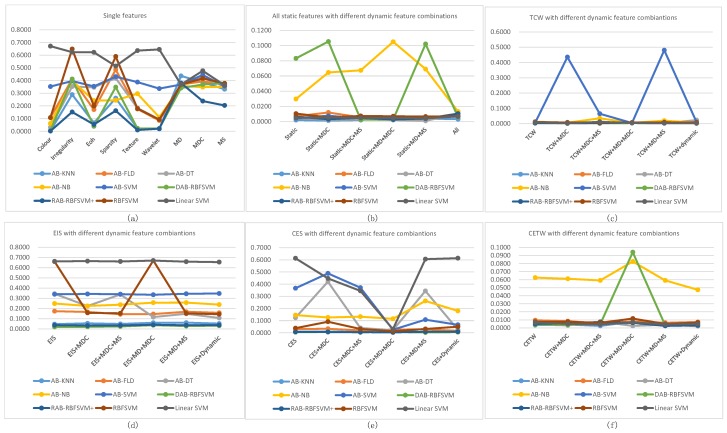
Generation errors of different classifiers with different feature datasets.

The proposed RAB-RBFSVM+ method outperforms all other methods tested as shown in Figure 12. It generates lower errors along with more stable performance for all feature combinations. Moreover, the proposed classifier with single static features gives better performance than single dynamic features in Figure 12a.

For further comparison, we test feature combinations with the proposed RAB-RBFSVM+ as shown in Figure 12b–f to find out which feature combination can achieve more satisfied performance for fire smoke detection. We list the experimental statistics in Table 6. The proposed detector using CES+MD+MS, TCW+MDC and TCW+MDC+MS feature combinations generate the three lowest errors. The feature combinations are chosen for application to fire smoke detection. Different videos with or without fire smoke objects are tested. For comparison, the following measures are considered,

(47) $$\mathrm{accuracy}=\frac{TP+TN}{N_{pos}+N_{neg}},$$

(48) $$\mathrm{detection}\;\mathrm{rate}=\frac{TP}{TP+FN},$$

(49) $$\mathrm{false}\;\mathrm{alarm}\;\mathrm{rate}=\frac{FP}{FP+TN},$$

where TP is the number of truth positives which gives the number of fire regions classified as fire, TN is the number of truth negatives, i.e., the number of non-fire regions which are classified as non-fire, FP is the number of false positives, i.e., the number of non-fire regions classified as fire, and FN is the number of false negatives, i.e., the number of fire regions classified as non-fire. $N_{p}os$ and $N_{n}eg$ are the total number of positives and negatives respectively. We compare the proposed method with different feature combinations and other two methods, [29,34], the rates described above are listed in Table 7. Our proposed method can achieve higher accuracy and detection rate along with lower false alarm rate as shown in Table 7.

**Figure 13:**
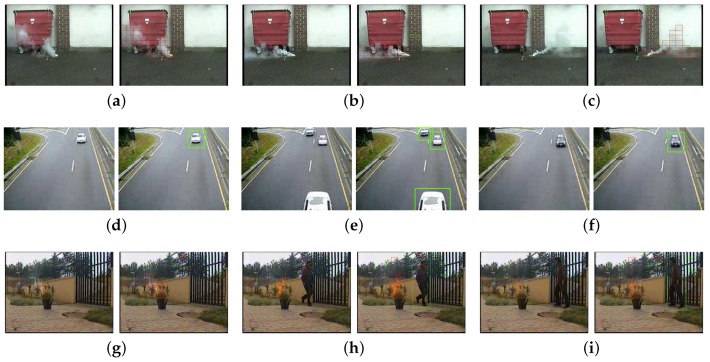
Detection results in three videos with or without smoke-fire. Smoke video with waste basket at frame (**a**) 121 (**b**) 166 and (**c**) 326. non-smoke video with cars on high way at frame (**d**) 11 (**e**) 26 and (**f**) 106. smoke-fire video with pedestrian at frame (**g**) 61 (**h**) 228 and (**i**) 244. Left image of each sub figure is the original frame image while the right one is the detected result.

Figure 13 shows the performance of our proposed detection method with TCW+MDC+MS features. Red boxes give the detected smoke or fire regions while green boxes give the motion regions without smoke or fire. There is no false smoke window detected by the proposed method in waste basket video and cars video, and small number of false smoke windows enclosing shaking trees in the last video due to the small size of these objects and low image quality of the noisy video.

## 5. Conclusions

This paper proposes a new fire smoke detector based on videos with Robust AdaBoost (RAB-RBFSVM) classifier. Features are extracted from different videos take by cameras. Static and dynamic features in different combinations are tested for fire smoke detection. The proposed classifier gives an efficient way of solving dilemma between errors and weights computing of AdaBoost. Further improvements including diversity and samples denoising for keeping balance of positive and negative training datasets are introduced to improve the performance of our detector. Tests with common datasets UCID and the extracted features from public video clips indicate that our proposed classifier outperforms the other classifiers. Extensive experiments on fire smoke detection are conducted and the results indicate effectiveness and performance advantages of our proposed fire smoke detector.

## Figures and Tables

**Table 1:** Literatures about fire smoke feature extraction.

	Color	Contour	Texture	Energy	Irregularity	Other	Dynamic	Training
[16]	*√*						*√*	
[17]	*√*			*√*			*√*	
[18]	*√*	*√*		*√*				
[15]	*√*			*√*	*√*			
[19]	*√*						*√*	
[20]	*√*						*√*	
[21]						Dark channel		
[22]						Sparsity		
[23]						Chrominance	*√*	*√*
[24]	*√*	*√*						*√*
[25]			*√*	*√*			*√*	*√*
[26]			*√*				*√*	*√*
[27]			*√*		*√*		*√*	*√*
[28]	*√*		*√*		*√*		*√*	*√*
[29]	*√*		*√*	*√*			*√*	*√*
Total	9	2	5	5	3	3	9	7

**Table 2:** ${\alpha_{m}}^{*}$ changes with different *n*.

*n*	${\alpha_{m}}^{*}\in$	${\alpha_{m}}^{*}\left(e_{m}=0.5\right)$
1	$\left[0.38,1\right]$	0.7191
3	$\left[0.05,1\right]$	0.3718
6	$\left[3.01\times 10^{-3},1\right]$	0.1383
9	$\left[1.65\times 10^{-4},1\right]$	0.0514
20	$\left[3.92\times 10^{-5},1\right]$	0.0001
100	$\left[9.27\times 10^{-43},1\right]$	$4.77\times 10^{-15}$
1000	$\left(0,\left.1\right]\right.$	0

**Table 3:** Errors of AdaBoost and Robust AdaBoost with different component classifiers (Bold numbers show the lowest errors of AdaBoost, Robust AdaBoost and classifier without boosting).

Base Classifier	AB				RAB				No Boosting
	*N* = 50	*N* = 100	*N* = 150	*N* = 200	*N* = 50	*N* = 100	*N* = 150	*N* = 200	
KNN	0.1346	0.1442	0.1346	0.1538	**0.0865**	0.1442	0.1538	0.1538	**0.0865**
Fisher	0.1635	0.1827	0.1923	**0.1442**	0.2019	0.2019	0.1923	0.1538	0.2596
Naive Bayes	0.1923	0.1635	**0.1538**	**0.1538**	0.1635	0.1731	0.1635	0.1923	0.2692
Decision Tree	0.1538	0.1635	0.1538	0.1538	**0.1346**	0.1538	0.1538	0.1442	0.2500
Linear SVM	0.2596	0.2788	0.6154	**0.2019**	**0.2019**	0.2212	**0.2019**	**0.2019**	0.2596
RBFSVM	0.5962	0.5962	0.5962	0.2308	**0.1058**	0.1442	0.1250	0.1250	0.2019

**Table 4:** Errors of RAB-RBFSVM and RAB-RBFSVM+ in different numbers of cycles.

Component Classifiers	RAB-RBFSVM	RAB-RBFSVM−	RAB-RBFSVM+
*N* = 50	0.1635	0.1154	0.0673
*N* = 100	0.1538	0.1731	0.0769
*N* = 200	0.1635	0.1250	0.0673
*N* = 300	0.1635	0.1346	0.0673
*N* = 400	0.0962	0.1346	0.0577
*N* = 500	0.1346	0.0962	0.0673

**Table 5:** Generation errors of different classifiers: AB-KNN, AB-FLD, AB-DT, AB-NB, AB-SVM, DAB-SVM, RAB-RBFSVM+ (For every single dataset, bold number gives the lowest error while underline number gives the second lowest error).

Datasets	AB-KNN	AB-FLD	AB-DT	AB-NB	AB-SVM	DAB-RBFSVM	RAB-RBFSVM+	RBFSVM	Linear SVM
Parkinsons	0.0928	0.2371	0.2680	0.3196	0.1753	0.1340	**0.0619**	0.2680	0.2577
Ionosphere	0.1657	0.2286	0.1314	0.1086	0.1771	0.1371	**0.0629**	0.1314	0.2000
Vote	0.0922	0.0737	0.0829	0.0645	0.0783	0.0645	**0.0599**	0.0737	**0.0599**
Sonar	0.1346	0.2212	0.1731	0.2212	0.2404	0.2019	**0.1346**	0.2019	0.2500
Wdbc	0.0704	0.0669	**0.0141**	0.0423	0.0634	0.0317	0.0282	0.0211	0.0317
Wpbc	0.4242	0.2626	0.2626	0.3232	**0.2424**	0.2525	0.2525	0.3232	**0.2424**
Mean	0.1400	0.1557	0.1332	0.1542	0.1396	0.1174	**0.0857**	0.1456	0.1488

**Table 6:** Errors of RAB-RBFSVM+ with different fire smoke features.

	CES	EIS	Static	TCW	CETW
+[ ]	0.0039	0.0193	0.0832	0.0056	0.0039
+MDC	0.0067	0.0207	0.1053	0.0014	0.0039
+MDC+MS	0.0032	0.0242	0.0025	**0.0011**	0.0046
+MD+MDC	0.0067	0.0362	0.0028	0.0032	0.0941
cc +MD+MS	0.0017	0.0277	0.1021	0.0018	0.0032
+Dynamic	0.0046	0.0316	0.0060	0.0018	0.0032

**Table 7:** Accuracy of our proposed method with different feature combinations, [29,34] (%).

Methods	Accuracy	Detection Rate	False Alarm Rate
Cai et al. [29]	74.10	92.70	7.30
Wu et al. [34]	73.41	94.48	5.52
AB-RBFSVM+ (with CES+MD+MS)	88.28	99.52	0.48
AB-RBFSVM+ (withTCW+MDC)	**93.31**	99.30	0.70
AB-RBFSVM+ (with TCW+MDC+MS)	91.25	**99.69**	**0.31**
